# Catheter-associated urinary infection in kidney post-transplant patients

**DOI:** 10.1590/S1516-31802001000500003

**Published:** 2001-09-01

**Authors:** Luiz Carlos de Oliveira, Antonio Marmo Lucon, Willian Carlos Nahas, Luiz Estevam Ianhez, Sami Arap

**Keywords:** Urinary infection, Urethral catheterization, Renal Transplantation, Infecção urinária, Cateterismo uretral, Transplante renal

## Abstract

**CONTEXT:**

There is still controversy as to the use and dosage of antimicrobial prophylaxis of the urinary infection associated with urethral catheterization in the post renal transplant period.

**OBJECTIVE:**

To determine whether patients develop urinary infection during short-term urethral catheterization after renal transplant without routine antimicrobial prophylaxis.

**DESIGN:**

Prospective study.

**SETTING:**

Kidney Transplantation Unit.

**SAMPLE:**

20 patients submitted to non-complicated kidney transplant, with a normal urinary tract and no risk factors present regarding urinary infection. Aged 15 to 65 years.

**MAIN MEASUREMENTS:**

Before the transplant, material from the urethral meatus and urine were collected for culture. After the transplant, in the period during which the patient was with short-term urethral catheterization (4 to 5 days), material from the urethral meatus and urine from the bladder and the collecting bag were taken daily from all recipients for culture.

**RESULTS:**

There was a predominance of coagulase-negative Staphylococcus and S. viridans in the normal urethral meatus flora and in the first two days of urethral catheterization. After the second day, there was a predominance of E. coli and E. faecalis. Urinary infection did not occur during the period of urethral catheterization. In the follow up only one female patient (7%) had asymptomatic bacteriuria caused by E.coli after the withdrawal of the urethral catheter.

**CONCLUSIONS:**

Infection urinary does not occur during the period of urethral catheterization in kidney post-transplant patients. Thus, antimicrobial prophylaxis is not recommended for these patients to prevent urinary infection.

## RESUMO

**CONTEXTO:**

Ainda existe controvérsia quanto ao uso e a forma de utilização de profilaxia antimicrobiana da infecção urinária associada ao cateterismo uretral no pós-transplante renal.

**OBJETIVO:**

Determinar se os pacientes desenvolvem infecção urinária durante o período de cateterismo uretral de curta duração após o transplante renal, sem usar a profilaxia antimicrobiana de rotina.

**TIPO DE ESTUDO:**

Estudo prospectivo.

**LOCAL:**

Unidade de Transplante Renal do Hospital das Clínicas, Universidade de São Paulo, São Paulo, Brasil.

**PACIENTES:**

20 pacientes submetidos a transplante renal não-complicado, com trato urinário normal e sem fatores de risco para infecção urinária. Idade variando de 15 a 65 anos de idade.

**VARIÁVEIS ESTUDADAS:**

Nos pacientes submetidos a transplante renal, durante o período de cateterismo uretral (4 a 5 dias), urina vesical, urina da bolsa coletora do sistema de drenagem fechado e material do meato uretral, foram coletados diariamente para exame de cultura.

**RESULTADOS:**

Houve um predomínio de bactérias *Staphylococcus sp* coagulase negativa e *S. viridans* na flora do meato uretral normal e nos primeiros dois dias de cateterismo uretral. Após o segundo dia de cateterismo uretral, houve predomínio de *E. coli* e *E. faecalis*. Infecção urinária não ocorreu durante o período de cateterismo uretral. No seguimento, apenas uma paciente (7%) desenvolveu bacteriúria assintomática causada por *E. coli* após a retirada do cateter uretral.

**CONCLUSÕES:**

Não ocorrem infecção urinária durante o período de cateterismo uretral no pós-transplante renal. Portanto, não há necessidade de profilaxia antimicrobiana nesses pacientes para prevenir a infecção urinária.

## INTRODUCTION

In hospitalized patients, urinary infection associated with urethral catheterization is common.^1,2^ It is the main source of nosocomial infection^1,3^ and septicemia by gram-negative bacteria.^4^ After renal transplant, urinary infection presents low morbidity,^5–8^ although it is the most frequent infection complication,^9–11^ the principal source of bacteremia^11,12^ and septicemia.^13^ There is still controversy as to the appropriate use and dosage of antibiotics in these patients.^5,9,14,15^

Both in Brazil and abroad, most papers published on the prophylaxis of urinary infection associated with urethral catheterization after renal transplantation have been based on studies where possible bias was not considered on analyses. There is no standardization regarding the duration of the urethral catheterization after surgery^9,15,16,17^ and often the studies only mention the occurrence of urinary infection when antibiotics are used^14–17^ for different periods of time.^9,14,15,17^

These facts led us to plan a prospective study such as might enable us better to understand the following aspects of urinary infection associated with urethral catheterization in post-transplant treatment: the incidence of positive culture in the bladder urine, in the urine from drainage system collecting bag and from a sample of the meatus during the period of urethral catheterization without the use of antibiotics; the clinical repercussion of occasional urinary infection associated with urethral catheterization; the clinical progress of patients with positive urinary culture who were asymptomatic and had not undergone any antibiotic treatment.

## METHODS

The procedures that follow were in accordance with ethical standards of the medical ethics committee of the Universidade de São Paulo.

### Patients and subjects

Forty-six kidney transplants were carried out between June 23, 1993, and April 3, 1994, in our Hospital. Of these, 28 patients were excluded: six with diabetes mellitus, six with abnormalities of the urinary tract, five children, three with surgical complications, three using antibiotics, one with morbid obesity, one with severe malnutrition, one with previous asymptomatic bacteriuria and one whose closed draining system had been violated. Thus, 18 adult patients with normal urinary tracts were selected during this period. Another two patients, with the same characteristics, underwent surgery in October 1994 and September 1995, bringing the total to 20 cases. The median age was 39.5 years. Eleven (55%) were female. There were 14 (70%) cadaver donor transplants and 6 (30%) living related donor transplants. Seven patients (35%) had no urine flow at the time of kidney transplantation. Thirteen patients (65%) had no history of urinary infection, five (25%) had had cystitis and two (10%) pyelonephrites. Eighteen patients (90%) had their original kidneys and two (10%) had already had nephrectomy. Nineteen patients (95%) had received their first transplant and one (5%) their second. The causes of chronic renal insufficiency is given in Table 1.

**Table 1:** Causes of chronic renal insufficiency in 20 patients who underwent renal transplants, in whom urinary infection associated with urethral catheterization was studied

*Causes of chronic renal insufficiency*	*N° of patients*	*(%)*
*Chronic glomerulonephritis*	*9*	*45*
*Primary malign nephrosclerosis*	*7*	*35*
*Polycystic renal disease*	*1*	*5*
*Renal amyloidosis*	*1*	*5*
*Chronic pyelonephritis*	*1*	*5*
*Unknown*	*1*	*5*
***Total***	***20***	***100***

### Study Procedures

In 13 patients with diuresis, material from the urethral meatus and urine were collected for culture and antibiogram on the day before surgery. In 7 patients without urine flow, material from the urethral meatus was collected for the same tests in the operating room. After that a Foley catheter was inserted immediately prior to surgery in all patients. In those patients without urine flow, the urine for tests were replaced by exams of the material collected through this urethral catheter by bladder washing with 20 ml of 0.9% NaCl. In the surgical technique of the renal transplantation the graft was placed in the iliac fossa, the renal vein was sutured by end-to-side anastomosis to the external iliac vein, and the arterial anastomosis was performed to the external or internal iliac artery. Urinary drainage was reestablished by the Gregoir et al.^18^ extravesical technique. Routine antibiotic prophylaxis was not undertaken in any of these cases.

Immunosuppression was initiated at the time of the transplantation in all the patients. Immunosuppression for recipients of kidneys from living donors consisted of prednisone and azathioprine. Recipients of kidneys from cadaver donors received prednisone, azathioprine and cyclosporin A. After transplantation, immunosuppression was gradually reduced to maintenance doses.^10^ After the transplant, in the period during which the patient was catheterized (4 to 5 days), material for culture and antibiogram from the urethral meatus, recently emitted urine (from the bladder) and urine from the collecting bag were collected daily. The collecting bag urine was taken through the bag tap. In the drainage of urine from the collecting bag sterilized gloves and recipients were not used. After the removal of the catheter, urine culture and susceptibility testing were performed on 14 patients weekly during a four-week follow-up period. Two cases of surgical complications and four of non-urinary infections were excluded.

## RESULTS

Table 2 presents the microorganisms found in the urethral meatus and the percentage of patients in whom they were found before catheterization on the 1^st^, 2^nd^, 3^rd^ and 4^th^ day of catheterization.

**Table 2:** Percentage of microorganisms found in the urethral meatus of 20 renal transplant patients before and during urethral catheterization

*Microorganisms*	*pre-cath* *(%)*	*1^st^ day* *(%)*	*2^nd^ day* *(%)*	*3^rd^ day* *(%)*	*4^th^ day* *(%)*
*Staphylococcus sp*	*60*	*30*	*35*	*35*	*60*
*S. viridans*	*30*	*10*	*20*		
*E. faecalis*	*15*	*15*	*10*	*30*	*35*
*E. coli*	*10*	*20*	*25*	*35*	*35*
*Corynebacterium sp*	*10*	*5*	*5*	*5*	
*Candida albicans*	*5*	*5*		*5*	*5*
*K. pneumoniae*	*5*				*5*
*P. mirabilis*	*5*		*5*	*5*	
*P. aeruginosa*	*5*				
*S. agalactiae*	*5*	*5*	*5*		
*Negative culture*	*5*	*25*	*25*		
*S. aureus*			*15*	*5*	*10*
*E. aerogenes*			*5*	*10*	
*M. morganii*					*5*

**Figure 1:**
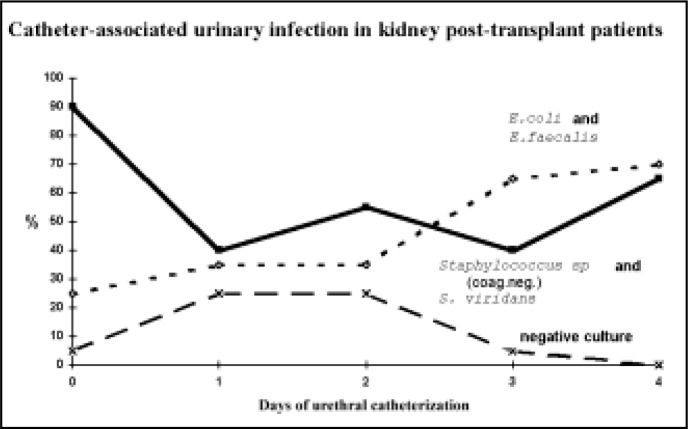
Study of the most frequently occurring bacteria in the culture of material from the urethral meatus before and in the first four days after urethral catheterization of the 20 renal transplant patients.

There was a predominance of two gram-positive bacteria in the urethral meatus flora, coagulase-negative *Staphylococcus* and *Streptococcus viridans* which occurred together in 90%, 40%, 55%, 40% and 65% of the patients, before catheterization and on the first, second, third and fourth days, respectively, of urethral catheterization. As for the gram-negative bacteria and *Enterococcus*, there was a predominance of *Escherichia coli* and *Enterococcus faecalis*, which occurred together in 25%, 35%, 35%, 65% and 70% of the patients before catheterization and on the first, second, third and fourth days, respectively, of urethral catheterization (Figure 1). One or two types of bacteria predominated in the urethral meatus flora both before catheterization and during the urethral catheterization period in which the incidence ranged from 70% to 90%.

There was no bacterial growth in the urine taken from the drainage system bag. Urinary infection did not occur during the period of urethral catheterization. After removing the urethral catheter from the 14 patients undergoing follow up, only one female patient (7%) had asymptomatic bacteriuria caused by *Escherichia coli* which was detected in the first week after the removal of the catheter and lasted for three weeks of study, and which after this period was successfully treated with trimethoprim-sulfamethoxazole.

## DISCUSSION

The control of the rejection and the prevention and treatment of infection are the major problems involved in successful renal transplantation.^10,19^ Parenteral antimicrobial prophylaxis of the urinary infection associated with urethral catheterization in the post renal transplant period has been employed by most transplant centers, through not by all.^5,6,8,15,20^ There is a world trend towards the reduction of the use of this prophylaxis. When used, it should be initiated immediately before the surgery and if continued should not be used for more than 24 hours after the transplant.^4,9,15^ Systemic antimicrobial prophylaxis has not been demonstrated to be of value for the prevention of this infection.^15^ Besides, immunosuppression does not damage the natural defenses of the host when it is used over a few days in the renal post-transplant period and the immune system plays no part either in the bladder colonization or in asymptomatic bacteriuria.^20,21^ The asymptomatic bacteriuria is by far the most frequent infection after renal transplantation and it is also one of the most benign infections unless it is associated with urological, surgical or serious immune complications.^5–8^ Thus, such prophylaxis should be aimed at protecting against wound infection, not against later urinary or other infections.^4,9,15^ If used, the antimicrobial prophylaxis of the urinary infection should not be initiated until the catheter has been removed.^15,22,23^ Children and patients with diabetes mellitus, abnormalities of the urinary tract, morbid obesity, severe malnutrition, surgical complications and violated closed drainage systems were excluded, because they were considered to be at greater risk for urinary or wound infections, sometimes with poorer recovery.^17,20,24,25^

There are other studies in which prophylactic antibiotics were not used routinely.^5,6,8,9,14,15,26^ Many authors agree that antimicrobial therapy should be given only after susceptibility testing in patients who present positive urine culture and symptoms at any time.^5,6,8,9,26^

Most urinary infections after kidney transplantation occur during the first month after the surgery.^5,6,8,11,17,26^ Thus, the patients were followed up until the fourth week after the removal of the urethral catheter in the post renal transplant period. In this study a urethral catheter remained in place for a median period of five days after the renal transplantation. This period of catheterization is longer than the one to four days mentioned in the literature.^6,9,26^ This longer period of urethral catheterization may cause greater harm to the normal urethral flora^27,28^ and may lead to a higher incidence of urinary infection.^5,24,28^

The gram-positive bacteria of the normal urethral meatus were present in 90% of the patients. Coagulase-negative *Staphylococcus* and *Streptococcus viridans* were by far the most frequent group of bacteria. The colonization of the urethral meatus by *Escherichia coli* and *Enterococcus faecalis* occurred in 25% of the patients. Studies have demonstrated that the longer the catheter is in place the greater the predominance of the gram-negative bacteria in the urethral flora.^27,28^ In our study, the group of *Escherichia coli* and *Enterococcus faecalis* started to predominate in the urethral meatus as from the second day of urethral catheterization and on the fourth day of catheterization their incidence reached 70%. In the first 24 hours of urethral catheterization, the presence of gram-positive bacteria was reduced from 90% to 40% and there was an increase in the percentage of negative culture of the material from urethral meatus from 5% to 25%. No data could be found in the literature as to the cause of this phenomenon, which is one of the most important factors in the pathogenesis of the urinary infection associated with urethral catheterization.^28–31^ The usage of the urethral catheter did not have any affect on the number of types of bacteria in the urethral meatus. Thus a 70% to 90% predominance of one or two types of bacteria, both before and during the period of urethral catheterization was demonstrated.

When the urethral catheters are removed within 1-4 days of the renal transplant, the development of urinary infection is unusual.^9,32^ In this study, urinary infection did not occur during the urethral catheterization period. In a group of 14 patients only one (7%), female patient had asymptomatic bacteriuria caused by *Escherichia coli* which began in the first week after the removal of the catheter and which persisted without treatment for three weeks, but with no clinical repercussion. After this period it was treated successfully with oral trimethoprimsulfamethoxazole for seven days. Many studies have shown that asymptomatic bacteriuria is an infection of a benign character.^5,6,7,8,10^

In none of the urine specimens taken from collecting bags of the closed drainage system could bacterial growth be demonstrated. Thus, when all due care was taken with the system of drainage, this latter prevented the rise of urinary infection by intraluminar route during the period after renal transplantation, in which the urethral catheterization lasted up to four days.

## CONCLUSIONS

Antimicrobial prophylaxis is not recommended for kidney post-transplant patients with normal urinary tract to prevent urinary infection.
